# Incidence and risk factors for febrile neutropenia in Japanese patients with non-Hodgkin B cell lymphoma receiving R-CHOP: 2-year experience in a single center (STOP FN in NHL 2)

**DOI:** 10.1007/s00520-019-04802-4

**Published:** 2019-05-15

**Authors:** Masahiro Yokoyama, Yoshiharu Kusano, Anna Nishihara, Norihito Inoue, Noriko Nishimura, Yuko Mishima, Yasuhito Terui, Tomoyuki Nukada, Takanobu Nomura, Kiyohiko Hatake

**Affiliations:** 1 Division of Hematology Oncology, The Cancer Institute Hospital, Japanese Foundation for Cancer Research, 3-8-31 Ariake, Koto-ku, Tokyo, 135-8550 Japan; 2 Medical Affairs, Kyowa Hakko Kirin Co., Ltd., Tokyo, Japan; 3 Department of Hematology, International University of Health and Welfare, Tokyo, Japan

**Keywords:** Febrile neutropenia, Incidence, Japan, Non-Hodgkin B cell lymphoma, R-CHOP, Risk factor

## Abstract

**Background:**

Myelosuppressive chemotherapy-induced febrile neutropenia (FN) is a life-threatening condition. Patients receiving granulocyte colony-stimulating factors (G-CSF) have shorter duration of neutropenia, faster recovery from fever, and shorter duration of antibiotics use. Most strategies for FN prevention using daily G-CSF and pegfilgrastim are based on overseas studies. Data on Japanese patients were lacking; thus, we previously determined the incidence of FN in non-Hodgkin B cell lymphoma (B-NHL) patients at our center. Here, we aimed to gain additional insights into pegfilgrastim use in this population.

**Methods:**

This single-center, retrospective, observational study (STOP FN in NHL 2) enrolled patients with B-NHL who underwent a regimen comprising rituximab and CHOP therapy over a 2-year period (January 2015–June 2017). The incidence of FN in cycle 1 of chemotherapy, risk factors for FN development, and use of daily G-CSF and pegfilgrastim were evaluated.

**Results:**

We evaluated 239 patients: 61 patients did not receive G-CSF and 178 received G-CSF. The incidence of FN was 10.5% (95% confidence interval [CI] 6.9–15.1%) in cycle 1 and 13.0% (95% CI 9.0–17.9%) in all cycles. The FN incidence was significantly lower (*P* = 0.0008) in patients receiving daily G-CSF and pegfilgrastim than patients not receiving G-CSF. Significant risk factors for FN were age ≥ 65 years, albumin < 3.5 g/dL, hemoglobin < 12 g/dL, and no prophylaxis with daily G-CSF/pegfilgrastim during cycle 1.

**Conclusions:**

The incidence of FN in cycle 1 and in all cycles and the identified risk factors were similar with those we previously reported; thus, our results validate previous findings.

**Trial registration:**

UMIN000029534.

**Electronic supplementary material:**

The online version of this article (10.1007/s00520-019-04802-4) contains supplementary material, which is available to authorized users.

## Background

Myelosuppressive chemotherapy-induced febrile neutropenia (FN) is a life-threatening condition associated with increased morbidity and mortality [1, 2] that often results in extended hospitalization and death [3]. FN may lead to unwanted chemotherapy dose reductions or may halt treatment altogether, which can compromise treatment outcomes [2].

According to the current Japanese guidelines [4], all patients who present an FN incidence ≥ 20% should receive prophylaxis granulocyte colony-stimulating factor (G-CSF), regardless of the presence or absence of risk factors, in order to prevent and treat FN induced by chemotherapy [5]. Further, all patients with risk factors for FN and FN incidence of 10 to < 20% should receive prophylaxis with G-CSF [4].

A recent meta-analysis of 30 randomized trials evaluated the relative efficacy of G-CSF products as primary prophylaxis for cancer patients receiving myelosuppressive chemotherapy. The meta-analysis concluded that the risk of FN was reduced with pegfilgrastim, filgrastim, lenograstim, and lipegfilgrastim when compared with no G-CSF or placebo. Although the use of filgrastim was heterogenous across the different trials, pegfilgrastim reduced FN risk compared with filgrastim (odds ratio = 0.61; 95% credible interval 0.40–0.98) [6]. Moreover, several trials have demonstrated that patients receiving G-CSF had a shorter duration of neutropenia, faster recovery from fever, and shorter duration of antibiotics use [7].

One cohort study identified that having non-Hodgkin lymphoma (NHL) and comorbidities such as anemia, HIV infection, and rheumatoid disease significantly increased the risk of developing FN [8]. Specifically, among patients receiving cyclophosphamide, doxorubicin, vincristine, and prednisone (CHOP) chemotherapy, characteristics including age ≥ 65 years, renal disease, cardiovascular disease, baseline hemoglobin < 12 g/dL, > 80% planned CHOP average relative dose-intensity, and no G-CSF prophylaxis were significantly associated with increased risk of FN [9]. However, the data obtained only corresponded to patients who received daily G-CSF, and there is no information on risk factors of FN in Japanese patients receiving pegfilgrastim. Identification of characteristics that predispose patients to FN who require treatment with these drugs will allow an optimal prophylactic use of G-CSF, thus improving chemotherapy delivery and patient outcomes [10].

Most strategies for FN prevention using daily G-CSF and pegfilgrastim are based on data from overseas clinical studies [11]. Additionally, the frequency of FN in clinical studies may differ from that in routine clinical practice. A recent systematic review reported that a 13% FN rate (95% confidence interval [CI] 8.7–17.9%) in a randomized clinical trial translated into a 20% FN rate in an observational study [12]. Therefore, it is very important to evaluate G-CSF use and G-CSF treatment outcomes in routine clinical practice in Japan in order to achieve effective FN prevention. Recently, we conducted a retrospective, observational study in a single center in Japanese patients with non-Hodgkin B cell lymphoma (B-NHL) and found that the incidence of FN was 9.1% (42 of 462 patients) in cycle 1 and 12.3% (57 of 462 patients) throughout all cycles, with 73.7% (42/57) of patients developing FN during cycle 1 [13].

Pegfilgrastim has a long half-life and is administered during the early phase of a treatment cycle, usually on the day following chemotherapy, and daily G-CSF is administered mostly to patients with signs of FN [14]. A lower dose (3.6 mg) of pegfilgrastim is used in Japan [4] compared with other countries (6 mg). However, our previous retrospective study in patients with B-NHL was conducted before pegfilgrastim became available in Japan [13], and no information was obtained on how both G-CSFs were actually administered in routine clinical practice. The present study aimed to gain additional insights into the use of pegfilgrastim, which had not been approved at the time of the previous study. Thus, we evaluated the incidence of FN in B-NHL patients who received rituximab and CHOP (R-CHOP regimen), identified risk factors for FN development by patient characteristics and FN onset, and evaluated the use of daily G-CSF and pegfilgrastim in routine clinical practice in Japan.

## Materials and methods

### Study design and treatment

In this single-center, retrospective, observational study (STOP FN in NHL 2), we retrospectively analyzed data from patients who underwent R-CHOP therapy over a 2-year period (between January 2015 and June 2017) at the Cancer Institute Hospital of the Japanese Foundation for Cancer Research, Tokyo, Japan.

This study was conducted in accordance with the Declaration of Helsinki and the Ethical Guidelines for Epidemiological Studies. The Ethical Review Board of our institute approved the study protocol. Based on those guidelines, informed consent from subjects was not required because the data analyzed were obtained from medical records. This trial was registered at the UMIN Clinical Trial Registry under the identifier UMIN000029534.

According to routine clinical practice in Japan, patients were hospitalized to receive cycle 1 of R-CHOP regimen. Whether patients received daily G-CSF or pegfilgrastim, as well as the corresponding dosing, was decided by each patient’s treating physician.

### Patients

Patients with malignant NHL who started and completed R-CHOP regimen during the study period, and patients who received at least three cycles of R-CHOP regimen were included in the study. Patients with HIV-related malignant lymphoma were excluded.

### Assessments

The investigational items related to the patients were age, sex, performance status, body mass index, characteristics of the disease (disease name, stage, and presence or absence of bone marrow infiltration), presence or absence of complications (diabetes mellitus, hepatic, renal or cardiac diseases, uncured wounds, and others), presence or absence of previous illness (surgery, infections, and FN within 1 month before initiation of the most recent R-CHOP regimen), and blood laboratory parameters (albumin, total bilirubin, hemoglobin, absolute neutrophil count [ANC], and absolute leukocyte count [ALC]). The investigational items related to the chemotherapy cycle were number of days to the next cycle and relative dose intensity (RDI), which represented the mean RDI for cyclophosphamide and doxorubicin and was calculated as follows: [(actual dose) / (planned dose)] / [(actual duration of treatment) / (planned duration of treatment)]. The investigational items related to the development of FN were body temperature, neutrophil count, prophylactic or therapeutic intervention (oral antibiotics, G-CSF, and treatment date from the initiation of chemotherapy), and whether or not hospitalization was required.

### Study endpoints

#### Primary endpoint

The incidence of FN in cycle 1 of chemotherapy was the primary endpoint. FN was defined as having an axillary temperature ≥ 37.5 °C and neutropenia with an ANC of < 500 μL or an ANC of < 1000/μL that is expected to decrease to < 500/μL within 48 h.

#### Secondary endpoints

The secondary endpoints were incidence of FN throughout all chemotherapy cycles, incidence of FN in cycle 1 of chemotherapy by patient demographic and clinical characteristics, risk factors involved in the onset of FN in cycle 1, relationship between the incidence of FN in cycle 1 and ALC, proportion of patients using antibiotics due to FN, proportions of patients with RDI ≥ 85% and RDI < 85%, and use of daily G-CSF and pegfilgrastim and their effects in routine clinical practice in Japan.

### Statistical analyses

The analysis set was defined as all patients enrolled in the study. Summary statistics such as the number of patients, mean, and standard deviation were calculated for continuous variables, and discrete variables were summarized by frequency. Continuous variables were examined using a *t* test or *u* test depending on the data distribution. Discrete variables were examined by chi-square test. The incidence of FN throughout all chemotherapy cycles was calculated as the percentage of patients who developed FN among all patients receiving R-CHOP regimen, and the corresponding 95% CIs were calculated.

No significance level was specified as this was not a hypothesis-testing study; however, a two-sided significance level of 5% was used for exploratory statistical analysis. We estimated the odds ratios in a logistic regression model. We then constructed a multivariate model based on the statistically relevant indicators (*P* < 0.10) found to influence the development of FN in a univariate model. These factors were then used in a stepwise variable analysis with an entry and removal probability of 0.20 to avoid overlooking factors affecting the development of FN. All statistical analyses were performed using SAS Versions 9.3 (SAS Institute, Inc., Cary, NC, USA).

### Simulation analysis

The simulation analysis has been previously described in detail [13]. Briefly, 1000 bootstrap samples were built by re-sampling from the population analyzed for the incidence of FN in cycle 1. For each bootstrap sample, the incidence of FN in cycle 1 without prophylaxis and with G-CSF was estimated for all patients included in the bootstrap samples. We obtained 1000 estimates of the incidence of FN in cycle 1 without prophylaxis and with G-CSF from the bootstrap samples. These estimates were then used to calculate the mean, which was used as the point estimate of the incidence of FN in cycle 1 without prophylaxis and with G-CSF for all patients included in the population analyzed for the incidence of FN in cycle 1. A CI was constructed based on the 2.5th and 97.5th percentiles of the estimated incidence of FN over 1000 bootstrapped samples. All analyses were performed without applying any imputation approach to deal with the missing data.

## Results

### Patient characteristics and treatment exposure

A total of 239 patients were evaluated, including 61 patients who were not treated with G-CSF and 178 patients who received G-CSF. Among 178 patients, 124 patients and 54 patients were administered daily G-CSF and pegfilgrastim, respectively.

Table 1 shows the main demographic and clinical characteristics of patients. Patients had a mean age of 64.0 ± 12.7 years and 46.9% were male. Most patients (95.8%) had an Eastern Cooperative Oncology Group performance status between 0 and 1, and the most common pathological diagnosis (66.9% of patients) was diffuse large B cell lymphoma.

**Table 1:** Demographic and clinical characteristics of patients in cycle 1

		*N* = 239
Age (years)		64.0 ± 12.7
Sex (male)		112 (46.9)
Body mass index (kg/m^2^)		22.6 ± 3.4
ECOG PS	0–1	229 (95.8)
	2–4	10 (4.2)
Pathological diagnosis	DLBCL	160 (66.9)
	Follicular lymphoma	41 (17.2)
	Transformed DLBCL	13 (5.4)
	Others	25 (10.5)
Stage	I–II	108 (45.2)
	III–IV	131 (54.8)
Bone marrow infiltration		29 (12.1)
Complications	Diabetes	21 (8.8)
	Hepatic or renal	13 (5.4)
	Other	127 (53.1)
Albumin (g/dL)		3.8 ± 0.6
Total bilirubin (mg/dL)		0.6 ± 0.3
Hemoglobin (g/dL)		12.5 ± 2.0
Absolute neutrophil count (× 10^9^/L)		4.2 ± 2.0
Absolute lymphocyte count (× 10^9^/L)		1.4 ± 1.4
Relative dose intensity (%)		88.7 ± 12.5

Data are presented as *n*(%) or mean ± SD

*ECOG PS*, Eastern Cooperative Oncology Group performance status; *DLBCL*, diffuse large B cell lymphoma; *SD*, standard deviation

The mean start date of daily G-CSF and pegfilgrastim treatment in cycle 1 was 10.18 ± 2.67 days and 2.59 ± 1.39 days, respectively, with the mean number of daily G-CSF dosing days of 2.98 ± 1.58 days. Online resource 1 shows the distribution of patients according to the day of G-CSF treatment start. In cycle 1, a small percentage of patients (2.4%) treated with daily G-CSF started treatment on days 0 to 3, while most patients (90.7%) treated with pegfilgrastim started treatment between days 0 and 3.

### Endpoints

For all patients (*N* = 239), the incidence of FN was 10.5% (95% CI 6.9–15.1%) in cycle 1 (primary endpoint) and 13.0% (95% CI 9.0–17.9%) in all cycles of chemotherapy.

**Fig. 1:**
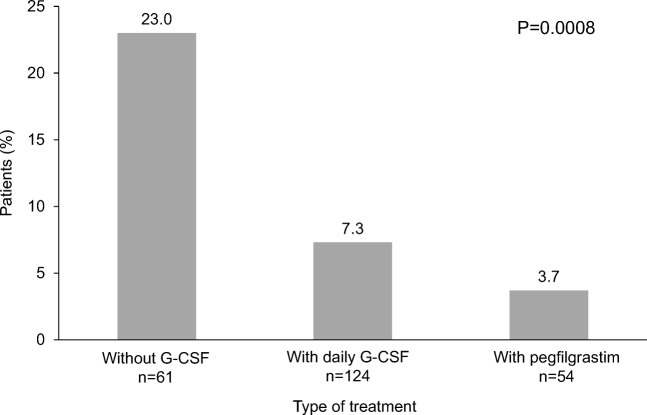
Incidence of febrile neutropenia in cycle 1 by type of treatment. *P* = 0.0008 for with daily G-CSF and with pegfilgrastim vs without G-CSF (chi-squared test). G-CSF, granulocyte colony-stimulating factor

The incidences of FN in cycle 1 were compared by type of treatment (i.e., without G-CSF treatment, with daily G-CSF and with pegfilgrastim). The incidence of FN was significantly lower in patients treated with daily G-CSF and pegfilgrastim, compared with patients without G-CSF treatment (7.3%, 3.7%, and 23.0%, respectively; *P* = 0.0008) (Fig. 1).

**Fig. 2:**
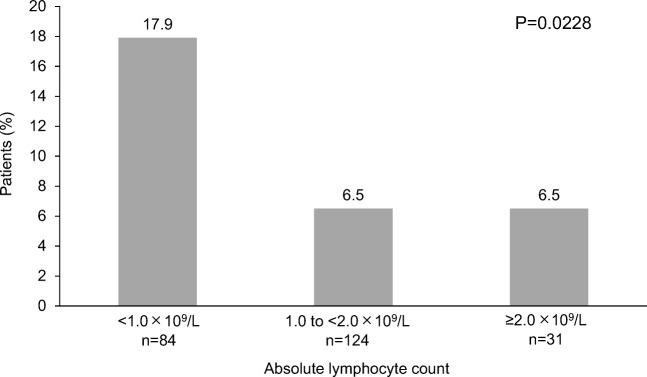
Incidence of febrile neutropenia in cycle 1 by absolute lymphocyte count levels. *P* = 0.0228 for absolute lymphocyte count < 1.0 × 10^9^/L vs 1.0 to 2.0 × 10^9^/L and > 2.0 × 10^9^/L (chi-squared test)

Table 2 shows the incidence of FN in cycle 1 stratified by patient characteristics. The incidence of FN was significantly higher in patients aged ≥ 65 years vs < 65 years, those with albumin < 3.5 g/dL vs ≥ 3.5 g/dL, those with hemoglobin < 12 g/dL vs ≥ 12 g/dL, those who used antibiotics vs those who did not used antibiotics, and those who did not use G-CSF in cycle 1 vs those who used G-CSF in cycle 1. There were no differences in the incidence of FN by sex, Eastern Cooperative Oncology Group performance status, body mass index, type of lymphoma, disease stage, presence or absence of bone marrow infiltration or complications, total bilirubin, RDI of cyclophosphamide or doxorubicin, ANC, or ALC. The proportion of patients with RDI ≥ 85% and RDI < 85% was 68.6% (164/239) and 31.4% (75/239), respectively. The incidences of FN in cycle 1 were compared by ALC levels (i.e., < 1.0 × 10^9^/L, 1.0 to < 2.0 × 10^9^/L, and ≥ 2.0 × 10^9^/L); there was a significantly higher incidence of FN in the group with ALC < 1.0 × 10^9^/L compared with the groups with ALC 1.0 to < 2.0 × 10^9^/L and ≥ 2.0 × 10^9^/L (17.9%, 6.5%, and 6.5%, respectively; *P* = 0.0228) (Fig. 2).

**Table 2:** Incidence of FN in cycle 1 by patient characteristics

Characteristic	*N* = 239 *n*/*N*	(%)	Chi-square (*P* value)
Age			
< 65 years	6/115	(5.2)	6.5051
≥ 65 years	19/124	(15.3)	(0.0108)^a^
Albumin			
<3.5 g/dL	14/53	(26.4)	18.5092
≥ 3.5 g/dL	11/186	(5.9)	(< 0.0001)^a^
Hemoglobin			
< 12 g/dL	19/86	(22.1)	19.4095
≥ 12 g/dL	6/153	(3.9)	(< 0.0001)^a^
Use of antibiotics			
No	1/156	(< 1)	46.2425
Yes	24/83	(28.9)	(< 0.0001)^a^
With or without G-CSF treatment in cycle 1 (treatment before development of FN)			
No	14/61	(23.0)	13.6431
Yes	11/178	(6.2)	(0.0002)^a^
Use of G-CSF in cycle 1 (treatment before development of FN)			
Without G-CSF	14/61	(23.0)	14.1505 (0.0008)^a^
With daily G-CSF	9/124	(7.3)	
With pegfilgrastim	2/54	(3.7)	

Parameters shown in the table are those with significant changes only. The incidence of FN was 10.5% (25 of 239) in cycle 1 and 13.0% (31 of 239) throughout all cycles. The majority of FN cases (80.6%; 25 of 31) occurred during cycle 1

*FN*, febrile neutropenia; *G-CSF*, granulocyte colony-stimulating factors

^a^Data were analyzed using chi-squared test

To identify the risk factors involved in the onset of FN in cycle 1, we conducted a univariate analysis of patient characteristics. The statistically and clinically relevant factors identified for cycle 1 were then used for a multivariate analysis (Table 3). Age ≥ 65 years, albumin < 3.5 g/dL, hemoglobin < 12 g/dL, and lack of prophylaxis with daily G-CSF and pegfilgrastim during cycle 1 were identified as significant risk factors for FN.

**Table 3:** Factors associated with the risk of febrile neutropenia in cycle 1 (univariate and multivariate analysis, *N* = 239)

	Univariate analysis			Multivariate analysis		
	OR	95% CI	*P* value	OR	95% CI	*P* value
Age (< 65 years, ≥ 65 years)	3.287	1.263, 8.551	0.0147	4.265	1.242, 14.639	0.0212
Sex (male, female)	1.366	0.587, 3.177	0.4688	–	–	–
ECOG PS (0–1, 2–4)	2.239	0.448, 11.183	0.3259	–	–	–
BMI (< 23 kg/m^2^, ≥ 23 kg/m^2^)	0.487	0.195, 1.215	0.1229	–	–	–
Pathological diagnosis (DLBCL, follicular lymphoma)	0.381	0.085, 1.705	0.2067	–	–	–
Pathological diagnosis (DLBCL, transformed DLBCL)	1.349	0.278, 6.557	0.7103	–	–	–
Pathological diagnosis (DLBCL, other)	0.645	0.141, 2.957	0.5728	–	–	–
Stage (I–II, III–IV)	1.864	0.771, 4.504	0.1665	–	–	–
Bone marrow infiltration (no, yes)	1.979	0.680, 5.759	0.2103	–	–	–
Diabetes mellitus (no, yes)	0.404	0.052, 3.148	0.3871	–	–	–
Hepatic or renal diseases (no, yes)	2.783	0.712, 10.874	0.1411	–	–	–
Albumin (≥ 3.5 g/dL, < 3.5 g/dL)	5.711	2.410, 13.531	< 0.0001	5.081	1.517, 17.015	0.0084
Total bilirubin (< 1 mg/dL, ≥ 1 mg/dL)	0.644	0.081, 5.144	0.6783			
Hemoglobin (≥ 12 g/dL, < 12 g/dL)	6.948	2.654, 18.186	< 0.0001	7.973	2.339, 27.179	0.0009
Absolute neutrophil count (≥ 2.69 × 10^9^/L, < 2.69 × 10^9^/L)	0.758	0.247, 2.322	0.6272	–	–	–
Absolute lymphocyte count (≥ 0.71 × 10^9^/L, < 0.71 × 10^9^/L)	2.605	1.071, 6.336	0.0347	–	–	–
Relative dose intensity (≥ 85%, < 85%)	1.528	0.652, 3.581	0.3290			
Prophylaxis with G-CSF (no, yes)	0.271	0.062, 1.188	0.0834			
Prophylaxis with daily G-CSF (no, yes)	0.263	0.106, 0.649	0.0037	0.059	0.015, 0.230	< 0.0001
Prophylaxis with pegfilgrastim (no, yes)	0.129	0.028, 0.598	0.0089	0.017	0.002, 0.119	< 0.0001

Although BMI and presence/absence of complications did not show statistical significance in univariate analysis, these were included in the multivariate analysis because they are considered clinically significant factors

*BMI*, body mass index; *CI*, confidence interval; *DLBCL*, diffuse large B-cell lymphoma; *ECOG PS*, Eastern Cooperative Oncology Group performance status; *FN*, febrile neutropenia; *G-CSF*, granulocyte colony-stimulating factors; *OR*, odds ratio

### Simulation analysis

As a result of the simulation analysis by multivariate analysis, four candidate risk factors for FN were identified (Table 4). Age ≥ 65 years, albumin < 3.5 g/dL, hemoglobin < 12 g/dL, and lack of prophylaxis with daily G-CSF or pegfilgrastim were identified as significant risk factors for the development of FN in cycle 1. These factors were then used to estimate the incidence of FN by multivariate logistic regression in patients without prophylactic administration of G-CSF. According to the simulation analysis, in patients who were not treated with G-CSF, the estimated incidence of FN in cycle 1 was 30.4% (95% CI 17.2–43.1%) (Table 4).

**Table 4:** Candidate risk factors for febrile neutropenia in cycle 1 (*N* = 239)

	Parameter estimate	Standard error	OR	95% CI	*P* value
Multivariate: stepwise, *P* = 0.20					
Age (< 65 years, ≥ 65 years)	1.4078	0.6438	4.087	1.157, 14.433	0.0288
Diabetes mellitus (no, yes)	− 1.9398	1.3622	0.144	0.010, 2.075	0.1544
Albumin (≥ 3.5 g/dL, < 3.5 g/dL)	1.6794	0.6266	5.362	1.570, 18.310	0.0074
Hemoglobin (≥ 12 g/dL, < 12 g/dL)	2.1435	0.6364	8.529	2.450, 29.692	0.0008
Prophylaxis with daily G-CSF (no, yes)	− 2.885	0.7033	0.056	0.014, 0.222	< 0.0001
Prophylaxis with pegfilgrastim (no, yes)	− 4.1641	0.9914	0.016	0.002, 0.109	< 0.0001
Estimated FN incidence, 30.4% (95% CI, 17.2–43.1%)					

*CI*, confidence interval; *FN*, febrile neutropenia; *G-CSF*, granulocyte colony-stimulating factors; *OR*, odds ratio

## Discussion

Data on the efficacy of daily G-CSF vs pegfilgrastim for the prevention of FN are limited, particularly in Japanese patients. To the best of our knowledge, this is the first study to evaluate the risk factors of FN in Japanese patients receiving daily G-CSF and pegfilgrastim in routine clinical practice. Although we had conducted a previous study that was limited to the evaluation of the effect of the prophylactic use of daily G-CSF and detection of risk factors for developing FN in cycle 1 in the same study center [13], the present study differs in that this study evaluated the data after pegfilgrastim became available in Japan and it includes subgroup analyses by the type of G-CSF used (daily G-CSF and pegfilgrastim) and treatment start date. Thus, we consider that the present results are a more accurate depiction of the use of G-CSF in clinical practice in Japan.

The incidence of FN in cycle 1 of chemotherapy (10.5%) and in all cycles (13.0%) was not different from that reported in our previous study (9.1% in cycle 1 and 12.3% in all cycles) [13]. Moreover, the incidence of FN in cycle 1 and timing of FN onset in Japanese patients with B-NHL reported herein is similar to that reported in previous studies [3, 8, 15, 16].

In the present study, an increase in the prophylactic use of G-CSF was observed, compared with the previous study [13]. This increase likely resulted from the implementation of the internal policy for pegfilgrastim use (Team G-LASTA), established based on the guidelines for use of G-CSF [4]. Both daily G-CSF and pegfilgrastim, administered for the prevention of FN, significantly reduced the incidence of FN compared with the group that did not receive G-CSF. Although no significant difference was observed between daily G-CSF and pegfilgrastim, the incidence of FN in patients treated with pegfilgrastim was numerically lower than that in patients treated with daily G-CSF. Additionally, we confirmed that the timing of daily G-CSF and pegfilgrastim administration (i.e., daily administration of G-CSF to patients with signs of FN and pegfilgrastim administration during the early phase of a chemotherapy cycle) in current clinical practice is comparable with what has been previously reported [14]. A possible reason why the incidence of FN was lower with pegfilgrastim than with daily-G-CSF is that daily G-CSF was administered later in the treatment cycle, when the signs of FN were confirmed, which may not be the optimal timing for G-CSF administration.

The presently identified risk factors for FN development in cycle 1 were similar to those reported previously [8, 9, 13]. No new risk factors for FN were identified, and thus, our results validate previous findings. This is valuable as it allows the identification of patients with a predisposition to develop FN and the implementation of an optimal prevention of FN with G-CSF in Japan.

Notably, patients with low ALC (< 1.0 × 10^9^/L) showed a significantly higher incidence of FN than other patients. This finding suggests that patients with low ALC could be more prone to developing FN; thus, these patients should be carefully monitored. Several previous studies have indicated that low baseline blood cell counts, including pretreatment ALC, are predisposing factors for FN development [15, 17–20].

In a study by Jenkins et al., the authors developed a predictive model to identify patients at increased risk of FN following chemotherapy based on pretreatment hematological indices [17]. Patients in the highest risk group (ALC ≤ 1.5 × 10^9^/L) had a 3.4-fold increased risk of developing FN (*P* = 0.001) and a 5.2-fold increased risk of cycle 1 FN (*P* < 0.001) [17]. In an earlier study by the same authors [18], the group of patients with the lowest values of ALC, which comprised 6% of the total population, had a risk of FN (21%) over fivefold greater than patients in the lowest risk group.

As a possible reason for such outcomes, Jenkins et al. proposed that low white blood cell counts, such as ALC, may reflect an insufficient bone marrow reserve. Relatively high bone marrow concentrations of the chemokine, stromal cell-derived factor 1 (SDF-1), promote retention of B lymphocyte and neutrophils in the marrow [21]. Thus, together with limiting amounts of endogenous G-CSF, increased SDF-1 levels in bone marrow may be another potential reason for low pretreatment ALC levels [18]. Additionally, patients with lower baseline ALC will likely have low B lymphocyte count as well, which may be a cause of the increased incidence of FN [21]. Another study reported that the incidence of FN was significantly higher in patients with lymphocyte counts ≤ 700/μL at chemotherapy day 5 (*P* = 0.0001), and a day 5 lymphocyte count ≤ 700/μL was identified as an independent risk factor for FN by logistic regression analysis [22].

In the guidelines, R-CHOP therapy is categorized as an intermediate risk for FN (with an FN incidence between 10 and 20%) [4]. The present study reported an incidence of 23.0%, which is categorized as high risk of developing FN (an FN incidence of 20% or greater), in patients not receiving G-CSF. Thus, the appropriate use of G-CSF seems relevant for preventing FN among patients receiving R-CHOP.

In the present study, the simulation analysis was done for reference. The resulting FN incidence was 30.4%, which is more than 10% higher compared with the result of the simulation analysis in the previous study (16.2%) [13]. However, the reason for the difference of nearly 10% in the incidence of FN in patients not receiving prophylactic G-CSF between the previous and current studies remains unclear. In the simulation analysis in the present study, we used the risk factors that were not identified in the previous study. The estimated odds ratio for hemoglobin was high, and there was a relatively strong relationship between hemoglobin and the use of G-CSF. In the group receiving G-CSF, compared with the group not receiving G-CSF, the proportion of patients with hemoglobin < 12 g/dL and albumin < 3.5 g/dL, who were at high risk for developing FN, was higher. Lower levels of hemoglobin and albumin may indicate a compromised systemic condition, which can further exacerbate the condition and lead to a higher incidence of FN. This could potentially explain a higher incidence of FN in the group receiving G-CSF compared with the group not receiving G-CSF.

This study has several limitations, such as those inherent to observational and retrospective studies. This study was conducted at a single center which hampers the generalizability of the results. As cycle 2 and subsequent cycles were administered on an outpatient basis, it is possible that the detection of FN was not as accurate as in cycle 1 (inpatient treatment). Further, the results may have been influenced by the medical care preferences (e.g., all patients were hospitalized during cycle 1) which are specific to routine clinical practice in Japan. The sample size was small, which may have precluded some results from reaching statistical significance. Finally, HIV-positive patients were excluded from the study because in Japan, HIV-positive patients are treated at specifically designated hospitals, and the study site was not one of these centers.

## Conclusions

In the present study, the incidence of FN in B-NHL patients who received R-CHOP regimen was 10.5% in cycle 1 of chemotherapy and 13.0% in all cycles. Additionally, age ≥ 65 years, albumin < 3.5 g/dL, hemoglobin < 12 g/dL, and lack of prophylaxis with daily G-CSF and pegfilgrastim were identified as significant risk factors of FN during cycle 1. Patients receiving daily G-CSF and pegfilgrastim had a significantly lower incidence of FN compared with those who did not receive G-CSF. The incidence of FN in patients receiving pegfilgrastim was lower than that in patients treated with daily G-CSF, although this difference was not statistically significant. A future large-scale randomized study should be performed to further validate the results of this small-scale retrospective study.

## Electronic supplementary material

ESM 1 (PPTX 45 kb)
