# Supplementary material for: Incidence and risk factors for febrile neutropenia in Japanese patients with non-Hodgkin B cell lymphoma receiving R-CHOP: 2-year experience in a single center (STOP FN in NHL 2)
Source: Support Care Cancer. 2019 May 15;28(2):571–9. doi: 10.1007/s00520-019-04802-4 (PMC6954143; doi:10.1007/s00520-019-04802-4)
Supplement: Supplementary file 1 — (PPTX 45 kb) [file 520_2019_4802_MOESM1_ESM.pptx]

## Slide 1
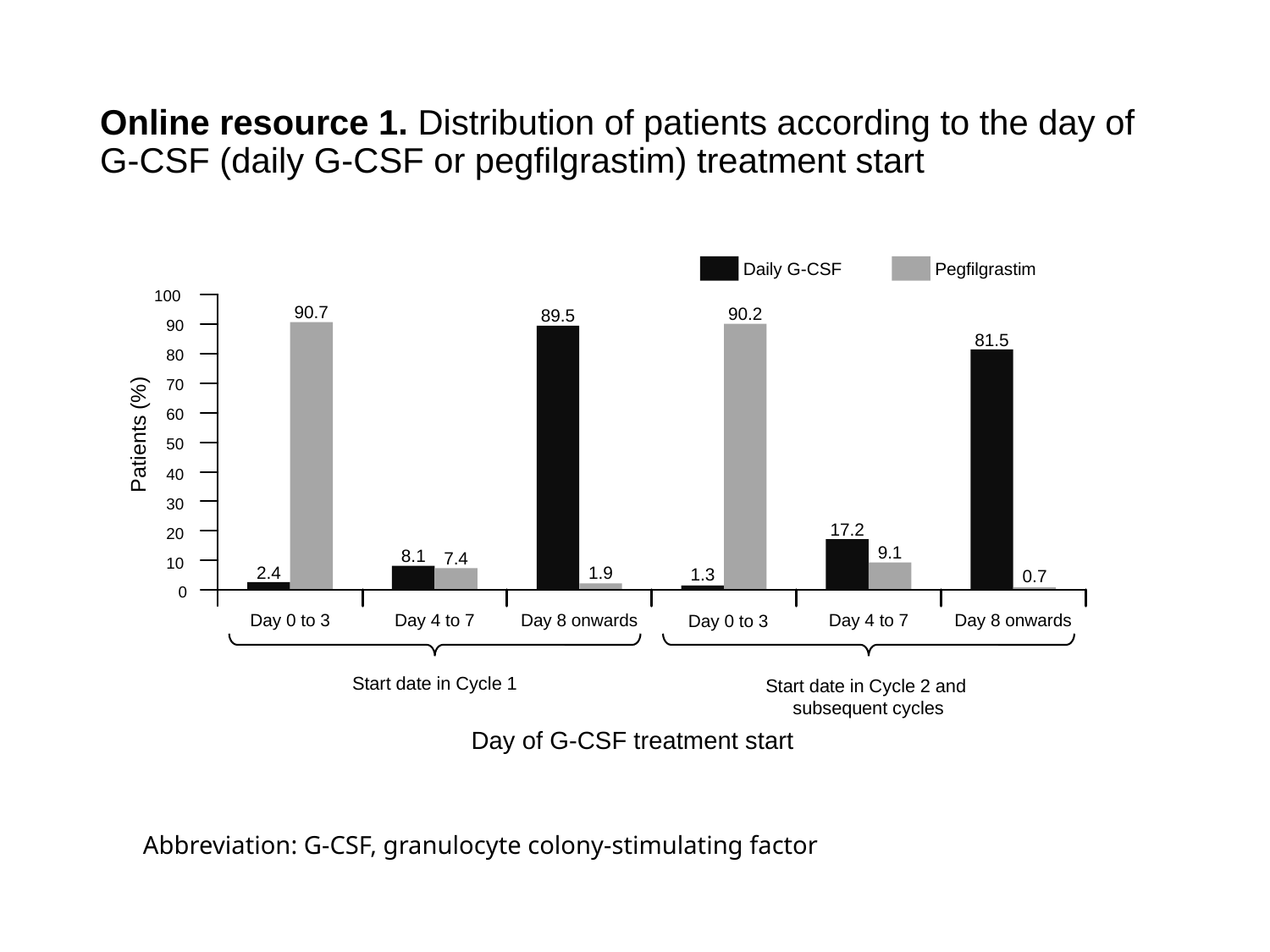

# Online resource 1. Distribution of patients according to the day of G-CSF (daily G-CSF or pegfilgrastim) treatment start
Daily G-CSF
Pegfilgrastim
100
90
80
70
60
50
40
30
20
10
0
90.7
90.2
89.5
81.5
Patients (%)
17.2
9.1
8.1
7.4
2.4
1.9
1.3
0.7
Day 0 to 3
Day 4 to 7
Day 8 onwards
Day 4 to 7
Day 8 onwards
Day 0 to 3
Start date in Cycle 1
Start date in Cycle 2 and
subsequent cycles
Day of G-CSF treatment start
Abbreviation: G-CSF, granulocyte colony-stimulating factor
